# Higher Elevations Tend to Have Higher Proportion of Plant Species With Glandular Trichomes

**DOI:** 10.3389/fpls.2021.632464

**Published:** 2021-04-12

**Authors:** Rui Wu, Simcha Lev-Yadun, Lu Sun, Hang Sun, Bo Song

**Affiliations:** ^1^Key Laboratory of Resource Biology and Biotechnology in Western China, Ministry of Education, College of Life Sciences, Northwest University, Xi’an, China; ^2^Key Laboratory for Plant Diversity and Biogeography of East Asia, Kunming Institute of Botany, Chinese Academy of Sciences, Kunming, China; ^3^Department of Biology and Environment, Faculty of Natural Sciences, University of Haifa at Oranim, Kiryat Tiv’on, Israel

**Keywords:** biotic interaction, elevation, glandular trichome, growth form, herbivorous insect, water availability, temperature

## Abstract

Glandular trichomes are well known to participate in plant chemical and physical defenses against herbivores, especially herbivorous insects. However, little is known about large-scale geographical patterns in glandular trichome occurrence. Herbivory pressure is thought to be higher at low elevations because of warmer and more stable climates. We therefore predicted a higher proportion of species with glandular trichomes at low elevations than at higher elevations. We compiled glandular trichome data (presence/absence) for 6,262 angiosperm species from the Hengduan Mountains (a global biodiversity hotspot in southwest China). We tested the elevational gradient (800–5,000 m a.s.l.) in the occurrence of plant species with glandular trichomes, and its correlations with biotic (occurrence of herbivorous insects) and abiotic factors, potentially shaping the elevational gradient in the occurrence of glandular trichomes. We found a significantly positive relationship between elevation and the occurrence of glandular trichomes, with the proportion of species having glandular trichomes increasing from 11.89% at 800 m a.s.l. to 17.92% at above 4,700 m. This cross-species relationship remained significant after accounting for phylogenetic relationships between species. Herbivorous insect richness peaked at mid-elevations and its association with the incidence of glandular trichomes was weak. Mean annual temperature was the most important factor associated negatively with glandular trichomes. Our results do not support the hypothesis that plant defenses decrease with increasing elevation. In contrast, a higher proportion of plant species with glandular trichome toward higher elevations is observed. Our results also highlight the importance of considering the simultaneous influences of biotic and abiotic factors in testing geographical variation in multifunctional plant defenses.

## Introduction

Geographical variation in species interactions (e.g., herbivory and predation) is widespread and is thought to generate concomitant patterns of species abundance, diversity, and functional traits (Thompson et al., 2005; Abdala-Roberts et al., 2016a; Descombes et al., 2017). Thus, studying the geographical patterns of biotic interactions is especially useful for our understanding of spatial variation in community composition within different environments (Schoonhoven et al., 2005). It has been widely believed that herbivory, one of the most common and important biotic (plant-animal) interactions, is more intense under warmer and more stable climates, found at lower elevations (Reynolds and Crossley, 1997; Metcalfe et al., 2014). In response to higher herbivory pressure, plants at lower elevations are predicted to invest more in their defenses (elevational herbivory defense hypothesis; Pellissier et al., 2014; Rasmann et al., 2014). Many studies have tested this hypothesis and have found a negative (Descombes et al., 2017), positive (e.g., Moreira et al., 2018), or no relationship between elevation and defense (Alonso-Amelot et al., 2007). Thus, despite the widespread interest in such a pattern, there is a disagreement about the direction of a general elevational gradient in plant defense.

There are several possible and reasonable reasons why the available evidence for elevational patterns in plant defense is mixed and unconvincing. First, because different defense-related traits may be effective against different herbivores, for example, spines mainly function in deterring large mammalian herbivores, while trichomes mostly deter insect herbivores (Barton, 2016). Different herbivore guilds with different life-histories usually respond in different ways to biotic or abiotic conditions and thus pooling various defense traits may gain contrasting results of elevational gradients in defense (Rasmann et al., 2014). Second, findings from several studies suggested that the tested elevational span was insufficient, and that studying only a relatively small number of species may also result in completely opposite results (e.g., Moles et al., 2011; Abdala-Roberts et al., 2016a). Third, if the data used in analyses originated from sampling with very different (and often inappropriate) methods, geographic pattern of a specific defense may be diluted (Moles et al., 2011), for example, variations in the extraction methodologies are suggested to influence the quantification of condensed tannins in leaves (Cork and Krockenberger, 1991). Fourth, plants with different growth forms might evolve different growth- and lifestyle-related defense strategies, ultimately influencing plant defense investment (Ronel and Lev-Yadun, 2012; Defossez et al., 2018; Xu et al., 2020). Unfortunately, variation in defenses among plant growth forms has frequently been overlooked or comparison of defenses among plant growth forms were limited by small datasets. Finally, it is well recognized that various defense traits respond not only to herbivore pressure, but could also be associated with abiotic conditions (Pratt et al., 2014) and resource availability (Abdala-Roberts et al., 2016a). For example, concentration of phenolics in leaves of *Quercus robur* increased independently with decreasing temperature toward higher elevations (Abdala-Roberts et al., 2016b). Thus, studies not considering the elevational variation in abiotic conditions may obscure the elevational gradients in plant defenses.

Indeed, overcoming the deficiencies mentioned above was very difficult in the past. For example, sampling including large number of plant species and spanning sufficient geographic breadth would be an enormously time- and effort-consuming enterprise. Now, these problems can be solved by compiling data from the literature and from data-bases. In this study, we used a consistent methodology to compare investment of a single but common and easily identified defense trait (i.e., glandular trichomes, a trait detailed described in the *Flora of China*) across a large number of plant species along a wide elevational range by collecting published literatures.

Glandular trichomes, an important and common trichome type, often produce, store, and secrete various secondary metabolites such as flavonoids, monoterpenes, or sesquiterpene lactones, and occur widely on many plant organs (Wagner, 1991). Although glandular trichomes may reflect an adaptation to various abiotic factors such as high-level O_3_ exposure, high levels of metal and non-metal ions in the soil, high vapor pressure deficit (Silva et al., 1996; Lihavainen et al., 2017; Li et al., 2018), one of their most important well-known roles is to serve as a defense against herbivores, especially insects (Dimock and Kennedy, 1983; Wagner, 1991; Hanley et al., 2007). Insect herbivores can be killed directly by ingested toxic exudates produced by glandular trichomes (Dimock and Kennedy, 1983; Liu et al., 2019). Some glandular trichomes can entrap insects arriving on plant surface by sticky exudates, and the entrapped insects usually die as a result of starvation or, in the case of small insect herbivores, of suffocation (Puterka et al., 2003; Simmons et al., 2004). Thus, glandular trichomes have attracted much attention from evolutionary ecologists who focus on how plants deter insect herbivores (e.g., Dimock and Kennedy, 1983; Kennedy and Sorenson, 1985; Ambrosio et al., 2008; Lev-Yadun, 2014; Liu et al., 2019). For example, both glandular trichome density and primary volatile organic compounds produced by glandular trichomes on leaf surfaces of *Ocimum gratissimum* plants increased significantly after being attacked by leaf-cutter ants (Tozin et al., 2017). Although we have numerous studies quantifying the geographic variation in glandular trichomes for single species (e.g., Horgan et al., 2009; Zhao et al., 2019), few studies have been conducted to test the roles of glandular trichomes in a large-scale and geographically diverse territory. These biogeographic patterns can advance our understanding of plant-herbivore interactions.

In this study, we tested the elevational variation in occurrence of glandular trichome in a large number of plant species and tested the association of the occurrence of glandular trichomes with both abiotic and biotic factors by compiling glandular trichome data (presence/absence) for 6,262 angiosperm species from the Hengduan Mountain region in southwestern China, ranging from 800 to 5,000 m a.s.l. Specifically, we asked: (1) is there an overall elevational gradient in the occurrence of plants with glandular trichomes, and does this gradient vary across growth forms? and (2) is there an association of abiotic and biotic factors with any such elevational variation in glandular trichomes?

## Materials and Methods

### Study Area

**FIGURE 1:**
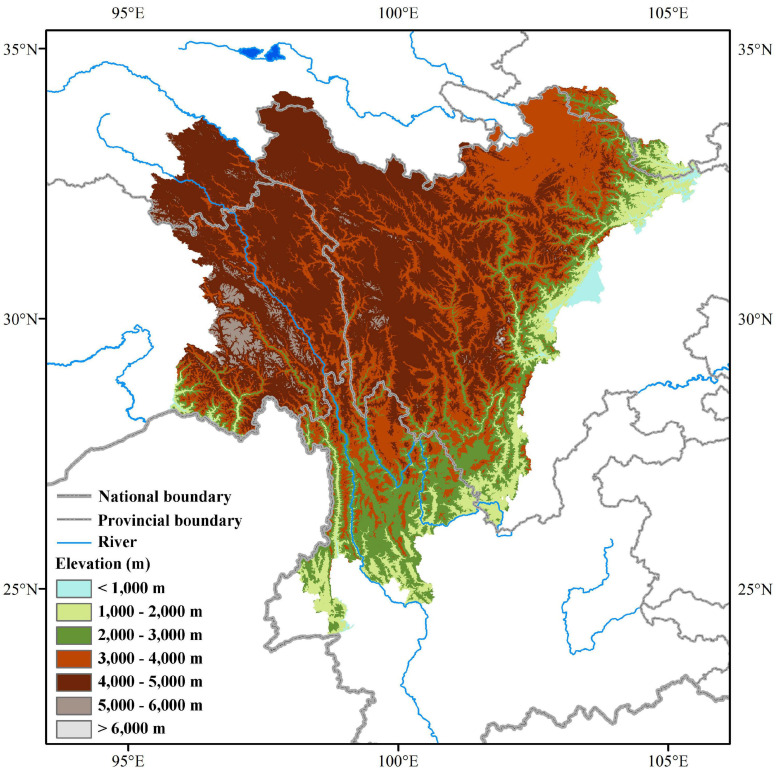
The location of the Hengduan Mountains in southwestern China.

This study covers the geographical region of the Hengduan Mountains in southwestern China, one of the 36 most important global biodiversity hotspots (Hrdina and Romportl, 2017). The study area includes northwestern Yunnan, western Sichuan, southeastern Tibet, southeastern Qinghai, and southern Gansu, stretching between 24°39′ to 34°20′ N, and 89°27′ to 104°36′ E, covering about 500,000 km^2^ (Figure 1). The average elevation drops from 4,000 to 5,000 m in the Western Sichuan and Eastern Tibet to 2,000 m in the Northwestern Yunnan. The climate in the Hengduan Mountains region is mainly influenced by the southeast monsoon and the plateau monsoon, with clear, dry winters, and warm, humid summers (Li and Zhang, 2010). It harbors diverse vegetation types, including dry valley scrub, broad-leaved forests, coniferous forests, alpine shrublands, meadows, and scree (Zhang et al., 2002).

### Data Collection

We compiled a list of all angiosperm species present in the study area, from the *Vascular Flora of Hengduan Mountains* (Wang, 1993), the *Flora of Yunnan* (Wu, 1977–2006), the *Flora of Sichuan* (Gao et al., 2003), the *Flora of Qinghai* (Liu, 1996), and the *Flora of Tibet* (Wu, 1983-1987). In order to determine the elevational range of each species included in our dataset, we downloaded all specimen information of the studied species collected from Hengduan Mountains region from the National Plant Specimen Resource Center^1^. Overall, our dataset included 147,150 specimens with elevation distribution ranging from 500 to 6,300 m. The elevation gradient between 500 and 6,300 m was divided into 100 m elevation intervals. Each species was considered as present at each 100 m belt between its upper and lower elevation limits (Bhattarai et al., 2004). This is a commonly used method (e.g., Vetaas and Grytnes, 2002; Bhattarai et al., 2004; Zhang et al., 2009; Bhatta et al., 2018), but it may result in an under-estimation of species richness at the extreme elevations or an over-estimation at mid-elevations (Vetaas and Grytnes, 2002). However, there is no reason to expect that any under- or over-estimation of species’ richness is likely to be biased in a way that would influence our estimation of the elevational gradient in the proportion of species with glandular trichomes. In order to determine whether elevational patterns of glandular trichomes was influenced by division of elevational intervals, the elevational gradients were also divided into 300 m and 500 m elevational intervals (also for herbivorous insect richness, mean annual temperature, and calculated plant-available water). In total, our dataset included 6,262 angiosperm species from 1,094 genera belonging to 175 plant families. We classified the species into one out of two growth form categories: herbaceous and woody plants (Galmán et al., 2018). Of the 6,262 species in our study, 4,010 were herbaceous (64.04%) and 2,252 were woody (35.96%). The proportion of herbaceous plants species increases with elevation, while the proportion of woody plants species decreases with elevation (Supplementary Figure 1).

Given that species with glandular trichomes must have trichomes, we began by scoring each species as having or not having any type of trichomes on any aboveground part of the plant over the entire life history according to the species’ descriptions in *Flora of China* (Wu et al., 1994–2012). Species with descriptions including the following terms were defined as having trichomes: pubescent, vill, bristle, puberul, toment, hair, pilose, sericeous, hirsute, setose, ciliate, strigose, velutinous, trichomes, lanate, hispid, wool, setulose, setae, fluff, and tomentellous. Species lacking any of these terms were sorted as not having trichomes. Then, we determined whether the trichomes of these species are glandular or not by checking the species descriptions in the *Flora of China* (Wu et al., 1994–2012) and specimens or communicating with experts. Although species may have intraspecific variation in density or phenotype of glandular trichomes within its range or between different development stages (Horgan et al., 2009), it is impossible to collect these data at our study scale. Thus, following the method of Moles et al. (2020), if a species was scored as having glandular trichomes, it was considered as having glandular trichomes at all elevations between its upper and lower limits and at various developmental stages.

It is impossible to gather species-specific data on insect herbivory at the huge scale of our analysis; thus, we used herbivorous insect richness as a proxy for herbivore pressure, due to their high correlation (Cardinale et al., 2006; Guimarães et al., 2014). We collected data on the elevational range for herbivorous insects in this area from *Insects of the Hengduan Mountains Region* (Chen, 1992) supplemented by personal communications with experts. A total of 2,979 insect species with known elevational distribution were included in the dataset, including beetles, moths, cicadas, and other groups of herbivorous insects. Although some insects may change their feeding habits across developmental stages, for example, larvae of some butterflies may be phytophagous, whereas their adults may be important pollinators, we considered them as herbivorous as long as they are phytophagous at any stages. Elevational ranges for each insect species were also interpolated using the same methods described above and divided into 100 m, 300 m, and 500 m elevational belts.

Mean annual temperature and mean annual precipitation are consistently considered to be correlated with species richness (Hawkins and Porter, 2003; Zhang et al., 2016). Thus, we download data of mean annual temperature (MAT; °C) from the WorldClim version2 database (Fick and Hijmans, 2017). Considering that the available water for plants (PAW; cm^3^/cm^3^), defined as the soil water between field capacity and permanent wilting point, is superior to precipitation in reflecting the water actually available for plants (Kirkham, 2005), we collected data on PAW from the global maps of soil hydraulic parameters (Zhang et al., 2018). Environmental layers at a 30-arcsecond resolution (∼1 km^2^) were projected into the same Albers Equal area coordinate system used for the study area. The longitude and latitude of each grid was obtained from the variable layer, and the corresponding elevation of each grid was obtained from the elevation layer using the longitude and latitude. The elevation and variable value of each grid were calculated, and the mean value of the data within each elevational belt of 100 m, 300 m, and 500 m was calculated to represent the variable value of this elevation belt.

### Data Analyses

In all analyses, the sampling unit was the each divided elevational belt. Because there was no adequate data about herbivorous insects for the elevational belts below 800 m and above 5,000 m, in combination with low plant species numbers within these elevational belts, only the elevational belts between 800 and 5,000 m were included in our analyses.

We used simple logistic regression to test the association between glandular trichome and elevation using “*glm*” function following Chen et al. (2017) and Pincheira-Donoso et al. (2017). For logistic regression analyses, glandular trichome data were modeled as a binary response variable (0 = absence of glandular trichome, 1 = presence of glandular trichome). That is, in all logistic regression models, the response variable was the number of species with glandular trichomes relative to the number of species without glandular trichomes in each elevational belt (Chen et al., 2017; Gao et al., 2020). Using the “*cbind*” function to combine the number of species with and without glandular trichomes in the response metric, our models weighted data points according to the number of species contained in elevational belt (Haas et al., 2018). Subsequently, to test whether the elevational gradient in incidence of species with glandular trichomes is related to growth form, multiple logistic regression was conducted where glandular trichome occurrence was predicted by elevation, growth form (herbaceous or woody plants) and their interaction.

Linear polynomial regression models were performed to test the elevational patterns of herbivorous insect richness, MAT and PAW as a function of elevation along the gradient. The best-fit models were selected by comparing models that included elevation as linear term or models that included elevation as both linear and quadratic terms based on the Akaike Information Criterion (AIC) value using “*AIC*” function (Supplementary Table 1). We performed simple logistic regression of incidence of glandular trichomes against each of the three factors (insect richness, MAT, and PAW) in order to examine the potential mechanisms of individual factors in explaining elevational gradients of glandular trichomes. Since no collinearity was found between these factors (Supplementary Table 2 and Supplementary Figure 2), we conducted multiple logistic regression analyses in order to explore the multivariate explanations for the elevational pattern of glandular trichomes, where the incidence of glandular trichomes was predicted by all biotic and abiotic variables (Hu et al., 2017).

In order to determine the relationship between glandular trichomes and elevation, herbivorous insect richness, mean annual temperature, and plant-available water in the context of phylogeny, phylogenetic logistic regression analyses were conducted with “*phylolm*” package (Ho and Ane, 2014). Phylogenetic logistic regression can be used to estimate parameter alpha and calculate likelihood of trait data. The parameter alpha, which reflects the overall rate of transition between binary states, which when close to zero corresponds to stronger phylogenetic effects (Ives and Garland, 2010). For these analyses, we generated a phylogenetic tree of study species using “*V.PhyloMaker*” package (Jin and Qian, 2019), which was implemented with a mega-tree derived primarily from Smith and Brown’s (2018) phylogeny. In addition, the elevation and the corresponding herbivorous insect richness, mean annual temperature, and plant-available water for each occurrence were extracted and the species’ mean values of all these variables were used (Chen et al., 2017; Gao et al., 2020).

Prior to the analysis, predictor variables were normalized using “*scale*” function to facilitate comparison of regression coefficients. For generalized linear models, the goodness-of-fit of each model was assessed by calculating McFadden’s *R*^2^ equivalent to *R*^2^ in ordinary least squares models (McFadden, 1974). All statistical analyses were conducted in the R (version 3.6.3) (R Development Core Team, 2020).

## Results

**FIGURE 2:**
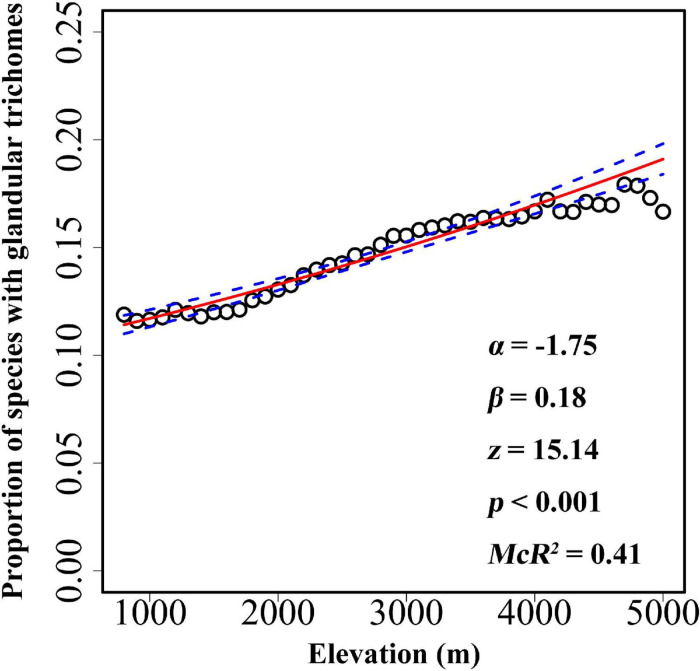
The relationships between glandular trichomes of plant species and elevation for every 100 m belt in the Hengduan Mountains region. The graph was visualized according to the proportion of plants with glandular trichomes, but the analysis was based on the binary data of presence versus absence of glandular trichomes. The fitted line (solid line) and estimated 95% confidence interval (dashed line) display the predicted probability of plants with glandular trichomes as fit by logistic regression models. α and β value was calculated after elevation being scaled.

The overall proportion of species with glandular trichomes in the Hengduan Mountains region was 14.1% (886 out of 6,262). The probability of finding species with glandular trichomes significantly increased toward higher elevations (Figure 2), ranging from 11.89% at 800 m a.s.l. to 17.92% at above 4,700 m. Multiple regression including elevation, growth form and their interaction indicated a significant effect of growth form on glandular trichomes (overall proportion of species with glandular trichomes: 15.6% for herbaceous species versus 11.6% for woody species). However, there was no significant interaction between growth form and elevation (Table 1), indicating that the relationship between glandular trichomes and elevation was not significantly different between species with different growth forms (Supplementary Figure 3).

**TABLE 1:** Logistic regression of elevation, growth form (herbaceous, woody), and their interaction against glandular trichome in the Hengduan Mountain region.

**Variable**	***z***	***p*-value**
Elevation	9.52	<0.01
Growth form	–4.60	<0.01
Elevation × growth form	1.236	0.22

**FIGURE 3:**
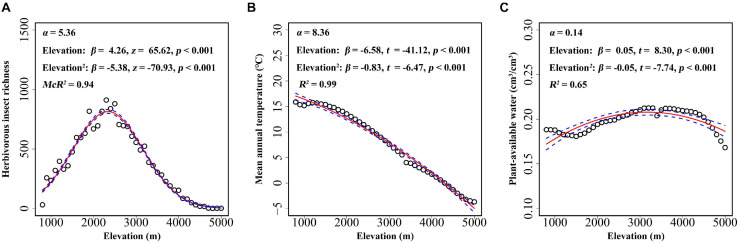
Relationship between elevation and herbivorous insect richness **(A)**, mean annual temperature **(B)**, and plant-available water **(C)** for every 100 m belt in the Hengduan Mountains region. Each point represents the value of herbivorous insect richness or the environmental variable in the corresponding elevation belt. The fitted lines (solid line) and estimated 95% confidence intervals (dashed line) were calculated using regression models that included elevation as both linear and quadratic terms. α and β value was calculated after elevation being scaled.

**FIGURE 4:**
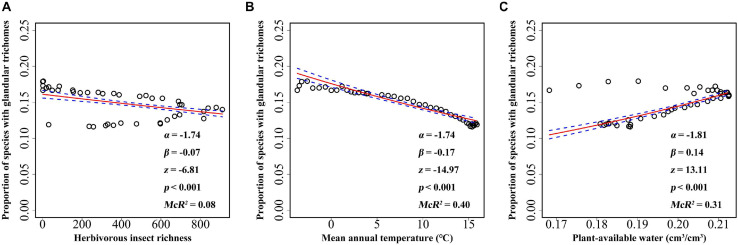
The relationship between glandular trichome of plant species and herbivorous insect richness **(A)**, mean annual temperature **(B)**, and plant-available water **(C)** for every 100 m belt in the Hengduan Mountains region. The graphs were visualized according to the proportion of plants with glandular trichomes, but the analysis was based on the binary data of presence versus absence of plants with glandular trichomes. The fitted lines (solid line) and estimated 95% confidence intervals (dashed line) display the predicted probability of plants with glandular trichomes as fit by logistic regression models. α and β value was calculated after predictor variables being scaled.

Elevational gradient in herbivorous insects’ species richness was unimodal, with the highest richness occurring at mid-elevations (between 2,000 and 3,000 m) (Figure 3A). Thus, insect herbivores did not show higher species richness at lower elevations. A significant association was found between mean annual temperature and elevation, with the mean annual temperature decreasing with increasing elevation (Figure 3B). Similar to herbivorous insect richness, plant-available water also showed a unimodal association with elevation, i.e., plants at mid-elevations having the highest water availability (Figure 3C). All factors, including biotic and abiotic variables, showed significant association with the incidence of plants with glandular trichomes when each predictor was considered alone (Figure 4). The strongest correlation was with annual mean temperature (*R*^2^ = 0.40), followed by the plant-available water (*R*^2^ = 0.31). Herbivorous insect richness showed a weak correction with the probability of finding plant species with glandular trichomes (*R*^2^ = 0.08). We also conducted analyses at 300 m or 500 m elevational intervals, and found that the main findings did not change (Supplementary Figures 4, 5).

When herbivorous insect richness, mean annual temperature, and plant-available water were considered together, the mean annual temperature was negatively associated, but plant-available water and herbivorous insect richness (marginally) were positively associated with the incidence of plant species with glandular trichomes (Table 2). The multiple logistic regression model explained 43.25% of the variation in the occurrence of plant species with glandular trichomes.

**TABLE 2:** Multiple regression models testing for the effects of biotic (HIR, herbivorous insect richness) and abiotic (MAT, mean annual temperature; PAW, plant-available water) on incidence of glandular trichomes in the Hengduan Mountains region.

**Predictors**	**β**	***z***	***p*-value**
HIR	0.03	1.98	0.048
MAT	–0.17	–6.71	<0.001
PAW	0.04	2.27	0.02

*β values were calculated after predictors being scaled.*

The incidence of species with glandular trichomes varied among families (Supplementary Figure 6), and the presence/absence of glandular trichomes was more phylogenetically conserved than being random (alpha = 0.02; Ives and Garland, 2010). Phylogenetic logistic regression analyses showed that the relationships between glandular trichomes and elevation and plant-available water remained significantly positive (*P* < 0.001; Supplementary Figure 7), and the relationships between glandular trichomes and herbivorous insect richness and mean annual temperature remained significantly negative (*P* < 0.001; Supplementary Figure 7). These results indicated that our main findings are unlikely to change when the evolutionary relationships among plant species were considered.

## Discussion

The question of how interactions between plants and herbivores varied along environmental gradients has interested ecologists for decades because of its importance for understanding mechanisms driving spatial variation in biodiversity and functional traits (Becerra, 2007). A long-held view in ecology is that plants from low elevations are better defended than are plants from high elevations (Schemske et al., 2009). Contrary to that prediction, our results showed that in the Hengduan Mountains region, plant species at higher elevations are more likely to have glandular trichomes even after accounting for the type of growth form and the evolutionary relationships between plant species, which is consistent with some previous studies (reviewed by Moreira et al., 2018). The congruence of these independent studies focusing on different defense traits strongly suggests that this widely accepted view needs to be reconsidered.

Defense investments are considered to reflect the historical herbivore pressure experienced by the plants, which is predicted to increase with decreasing elevations, due to the more benign and stable climate (Pellissier et al., 2012; Raffa et al., 2013). This served as the foundation for the view that low-elevation plants are thought to be better defended than are plants from higher elevations (Rasmann et al., 2014). Although we do not have direct data on herbivory pressures from insect herbivores experienced by the plants in our study area, many studies have shown that herbivorous insect richness could be used as a measure of herbivory pressure, since these two things are highly correlated (Cardinale et al., 2006; Guimarães et al., 2014). In accordance with many other studies of herbivore pressure along elevational gradients (reviewed by Moreira et al., 2018), in the Hengduan Mountains region, herbivorous insect richness was not the highest at low elevations, but rather peaked at mid elevations, which means that there is no higher selection pressure for higher defense against herbivorous insects at lower elevations. Thus, it is not surprising that plants at lower elevations did not have the highest probability of having glandular trichomes.

Interestingly, although herbivorous insect richness varied along elevational gradients (i.e., peak at mid-elevation), elevational variation in herbivory pressure did not result in concomitant gradients in the proportion of plants with glandular trichomes. Furthermore, our univariate regression showed a negative, albeit weak (*R^2^* = 0.08), relationship between herbivorous insect richness and incidence of species with glandular trichomes along the elevational gradients. In addition to herbivorous insects, glandular trichomes have also been found to function in protecting against mollusks (e.g., Westerbergh and Nyberg, 1995; Tomas et al., 2019). Unfortunately, we did not have herbivory or distribution data for mollusks in our study areas. Studies conducted at other sites have found that gastropod diversity generally decreased with increasing elevation (e.g., Liew et al., 2010; Baur et al., 2014; Schmera and Baur, 2014). If such pattern is true for the Hengduan Mountains region, higher proportion of species with glandular trichomes at higher elevations is unlikely to relate to herbivory pressure from mollusks in our study area. Our results indicated that considering only herbivory pressure by herbivores is insufficient to explain geographic variation in plant defenses in general (e.g., Abdala-Roberts et al., 2016a; Tindall et al., 2016; Moles et al., 2020), or the occurrence of glandular trichomes in particular.

Recent work has emphasized the importance of considering abiotic factors in testing for geographic variations in interactions between plants and herbivores (Endara and Coley, 2011; De Long et al., 2015; Zhang et al., 2016). In this study, we analyzed the effects of multiple abiotic factors associated with elevational gradients in plant traits and herbivorous insect richness on the incidence of glandular trichomes, and found that abiotic factors significantly better explained the variation (e.g., 40% and 31% for mean annual temperature and plant-available water, respectively) than herbivorous insect richness (8%). The proportion of plant species with glandular trichomes increases with decreasing temperature and with increasing water availability. Furthermore, after accounting for herbivorous insect richness in the multiple regression, mean annual temperature, and plant-available water remained significantly correlated with the occurrence of glandular trichomes. Plants growing in stressful habitats generally grow more slowly, and may adopt higher levels of resistance to herbivores (growth-defense trade-off hypothesis; Herms and Mattson, 1992), because the cost of losing tissues in these habitats is relatively higher (e.g., Coley et al., 1985). This can explain the negative correlation between incidence of plant species with glandular trichomes and mean annual temperature. Production of glandular trichomes is costly, for example, in some plant species, production of glandular trichomes has been documented to be limited by soil water content (e.g., Lauter and Munns, 1986; Elle et al., 1999). This can partly explain the positive correlation of incidence of glandular trichomes with plant-available water found in our study. However, compared with plant-available water, mean annual temperature explained 30% more of the variation in the incidence of species with glandular trichomes. Thus, investing more resources in defense is favored under more stressful conditions (i.e., lower temperature found at higher elevations in this study; Janzen, 1974; Coley et al., 1985).

It is important to note that many plant traits have dual roles in protection against herbivores and environmental stressors (Moles et al., 2011). For example, in addition to protecting from herbivory, it was found that glandular trichomes play an important role in protecting plants against intense UV radiation by accumulating flavonoids (Tattini et al., 2000). Unfortunately, we cannot get data on UV radiation with sufficient high resolution along the elevational gradients in our study area. However, UV radiation has long been confirmed to increase with increasing elevation due to smaller UV absorbing air mass (Körner, 2003). In response to higher UV radiation, plants at high elevations should have a higher level of defenses against UV radiation than plants at low elevations (Körner, 2003; Song et al., 2020), which may also partly explain the positive correlation between the incidence of species with glandular trichomes and elevation observed in this study. Thus, in order to improve our understanding of the underlying mechanisms associated with elevational variation in the occurrence of glandular trichomes, further studies should be conducted using manipulative experiments along elevational gradients, after controlling for other relevant factors.

Some intra-specific studies have found that glandular trichomes (i.e., density and composition) may shift within a species’ range (e.g., Horgan et al., 2009). Unfortunately, our study cannot account for this because we do not have corresponding data. However, as a parallel line of research, inter-specific comparisons across broad spatial, and multi-species scale are necessary for uncovering the macro-evolutionary patterns in plant defenses, which is especially important for understanding the elevational patterns of plant-herbivore interactions and the resultant elevational diversity gradients (Futuyma and Agrawal, 2009; Schemske et al., 2009). Thus, although our analyses were based on simplified binary data (presence/absence of glandular trichomes), we have been able to provide important insights into macro-evolutionary patterns in investments of glandular trichomes by plants along elevational gradients in the Hengduan Mountains region. In order to better understand the underlying mechanisms driving macro-ecological patterns of glandular trichomes in this region, further studies assessing geographic patterns of plant intra-specific variation should be conducted.

## Conclusion

In one of the largest empirical study of a single defense trait along elevational gradients to date, with more than 6,000 plant species and spanning 4,200 m of elevation, we found that plants from higher elevations had higher levels of defense against herbivores *via* glandular trichomes than did low-elevation plants, a finding that is contrary to the prevailing view on this topic (e.g., Rasmann et al., 2014). In addition to contributing important data to the current debate on the generality of the elevational herbivory defense hypothesis, our results highlight the importance of considering the simultaneous effects of abiotic drivers in understanding geographic variation in plant defenses (or any other traits), that have usually been overlooked (Abdala-Roberts et al., 2016a).

## Data Availability Statement

The raw data supporting the conclusions of this article will be made available by the authors, without undue reservation.

## Author Contributions

RW collected the data. RW and LS performed the analyses. BS and HS conceived the original research plan. BS, RW, and SL-Y wrote the article. All authors read and approved the final manuscript.

## Conflict of Interest

The authors declare that the research was conducted in the absence of any commercial or financial relationships that could be construed as a potential conflict of interest.

## Footnotes

1 www.cvh.ac.cn
