# Supplementary material for: Higher Elevations Tend to Have Higher Proportion of Plant Species With Glandular Trichomes
Source: Front Plant Sci. 2021 Apr 12;12:632464. doi: 10.3389/fpls.2021.632464 (PMC8075162; doi:10.3389/fpls.2021.632464)
Supplement: Supplementary file 1 [file Data_Sheet_1.docx]

Higher elevations tend to have higher proportion of plant species with glandular trichomes

Rui Wu, Simcha Lev-Yadun, Lu Sun, Hang Sun, Bo Song

**Table S1** Details of models including elevation as linear term or including elevation as both linear and quadratic terms.

| Model | AIC value |
| --- | --- |
| HIR ~ Elevation | 8415 |
| HIR ~ Elevation + Elevation^2 | 663 |
| MAT ~ Elevation | 129 |
| MAT ~ Elevation + Elevation^2 | 100 |
| PAW ~ Elevation | -257 |
| PAW ~ Elevation + Elevation^2 | -294 |

HIR：herbivory insect richness, MAT: mean annual temperature, PAW: plant-available water.

**Table S2** VIF (variance inflation factor) matrix of variables. HIR = herbivorous insect richness, MAT = mean annual temperature, PAW = plant-available water.

| variables | HIR | MAT | PAW |
| --- | --- | --- | --- |
| VIF | 2.12 | 4.00 | 2.55 |


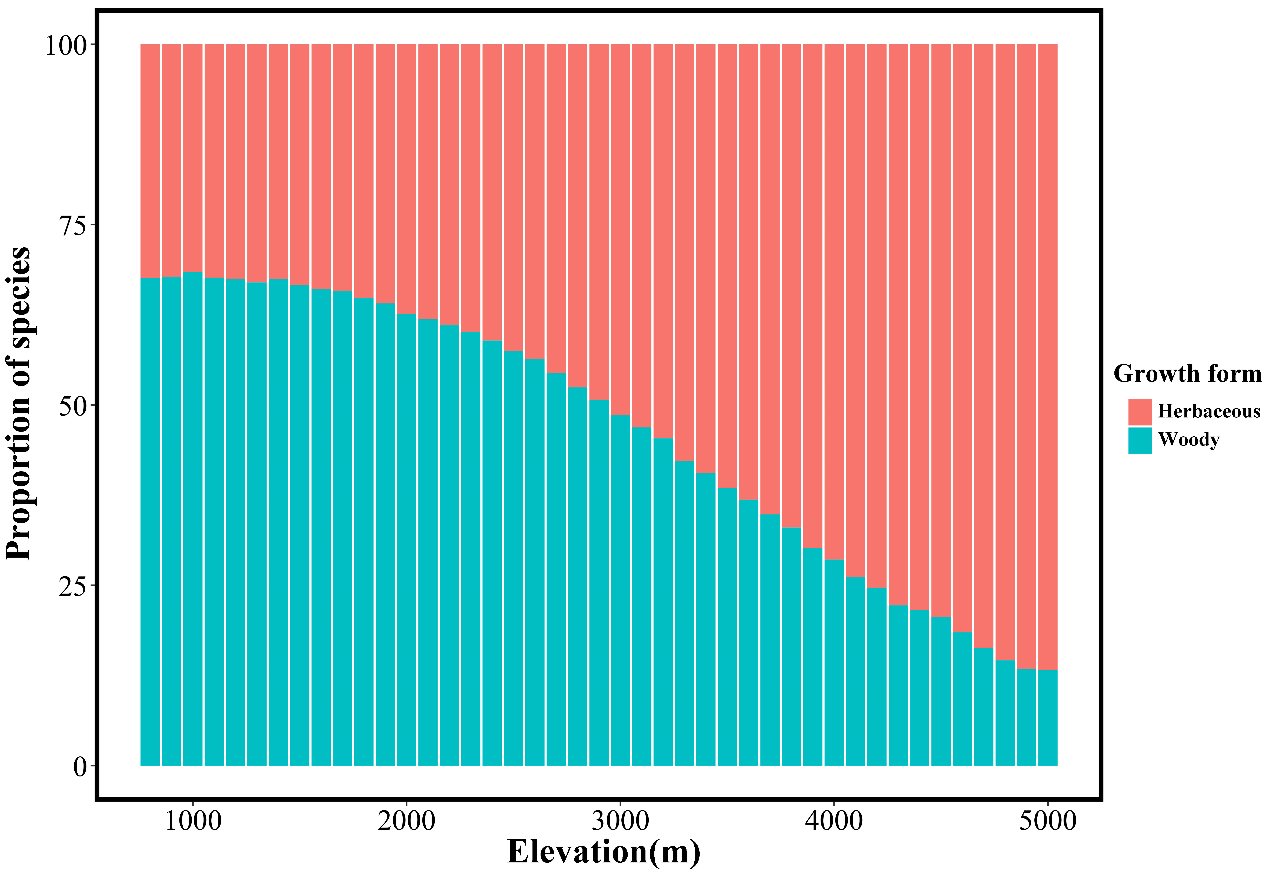


**Fig. S1** Growth form spectra along elevational gradients. The elevational ranges are from 800 to 5,000 m a.s.l.


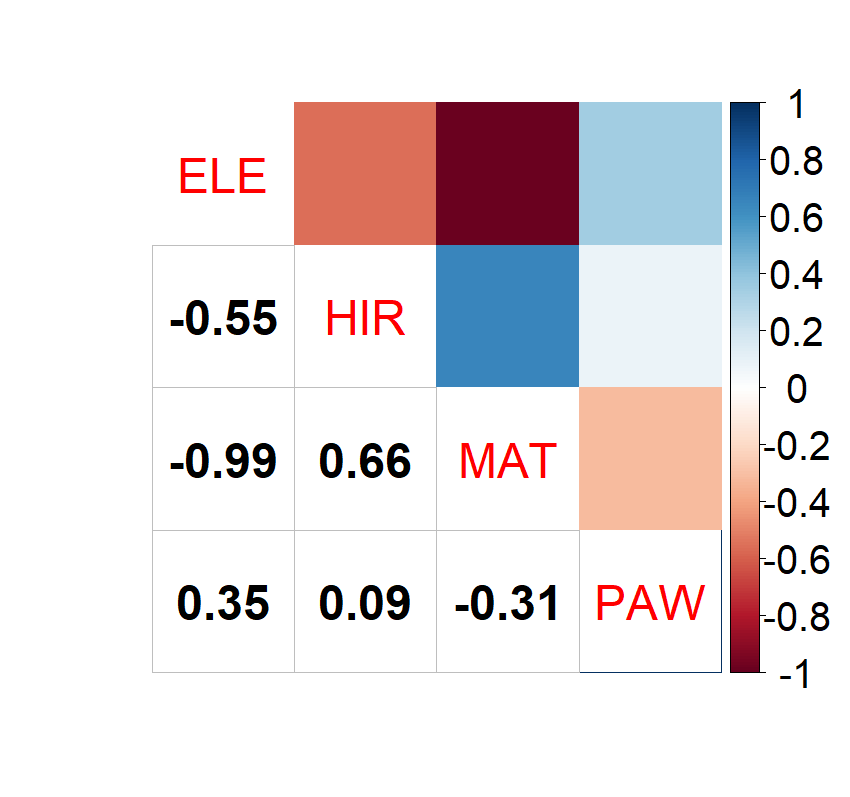


**Fig. S2** The correlation matrix of the Pearson’s correlation coefficients between variables. Positive correlations are displayed in blue and negative correlations in red. Colour intensity and circle size are proportional to the Pearson’s correlation coefficients. The legend on the right side of the correlogram shows the correlation coefficients and the corresponding colours. ELE =elevation, HIR= herbivorous insect richness, MAT = mean annual temperature, PAW = plant-available water.

**
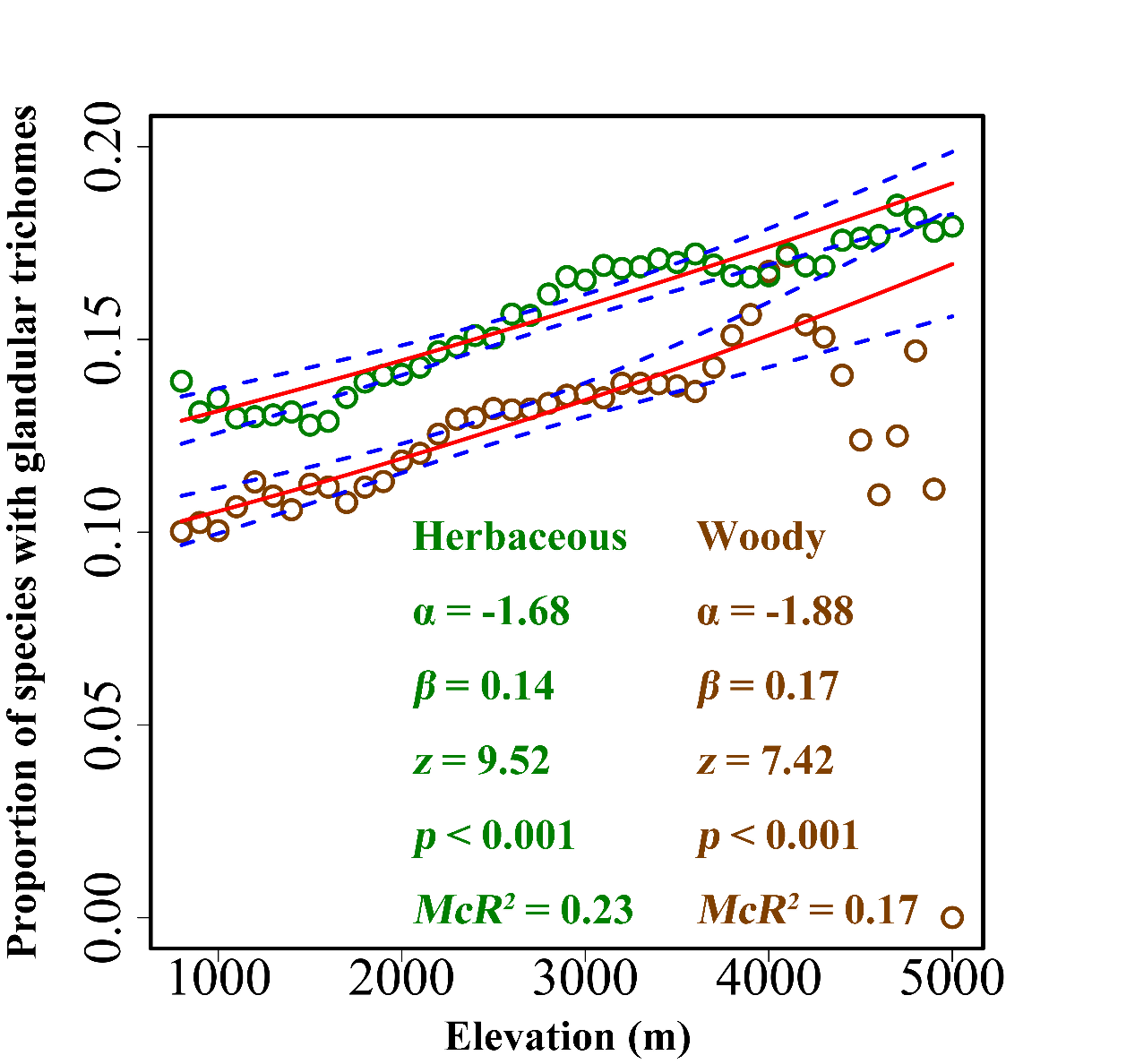
**

**Fig. S3** The relationships between glandular trichomes and elevation for every 100 m belt in the Hengduan Mountains region. Herbaceous species are indicated in green and woody species are indicated in brown. The graph was visualized according to the proportion of plants with glandular trichomes, but the analyses were based on the binary data of presence *versus* absence of glandular trichome. The fitted lines (solid line) and estimated 95% confidence intervals (dashed line) display the predicted probability of plants with glandular trichome as fit by logistic regression models.*α* and *β* values were calculated after elevation being scaled.


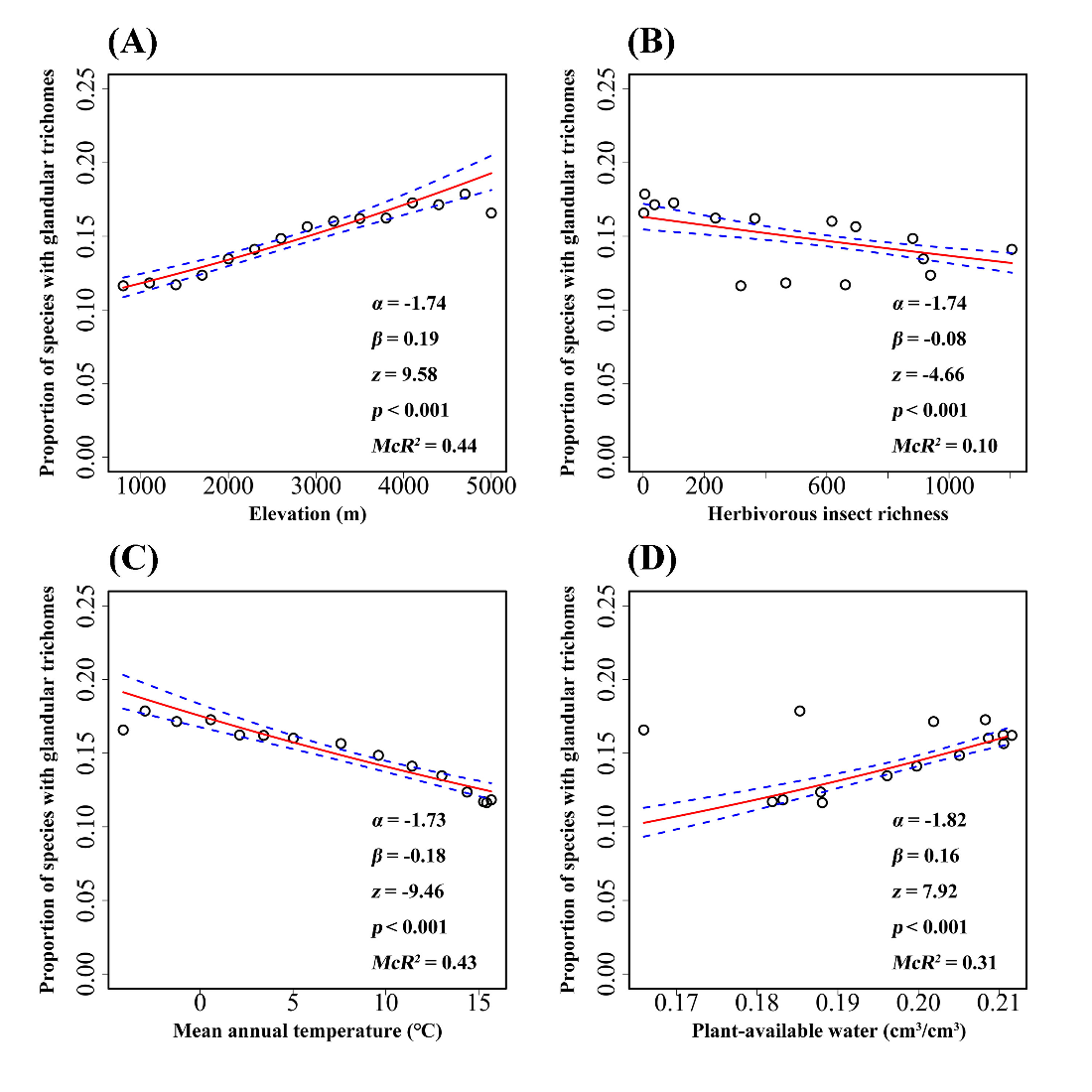


**Fig. S4** Relationship between glandular trichomes and elevation (A), herbivorous insect richness(B), mean annual temperature (C) and plant-available water (D) in the Hengduan Mountains region. The graphs were visualized according to the proportion of plants with glandular trichomes, but the analyses were based on the binary data of presence versus absence of glandular trichomes. Each point represents the value of proportion of plants with glandular trichomes in the corresponding elevation belt. The fitted lines (solid line) and estimated 95% confidence intervals (dashed line) display the predicted probability of plants with glandular trichome as fit by logistic regression models. The elevational ranges are from 800 to 5,000 m with every 300 m belt. *α*and *β* values were calculated after predictors being scaled.


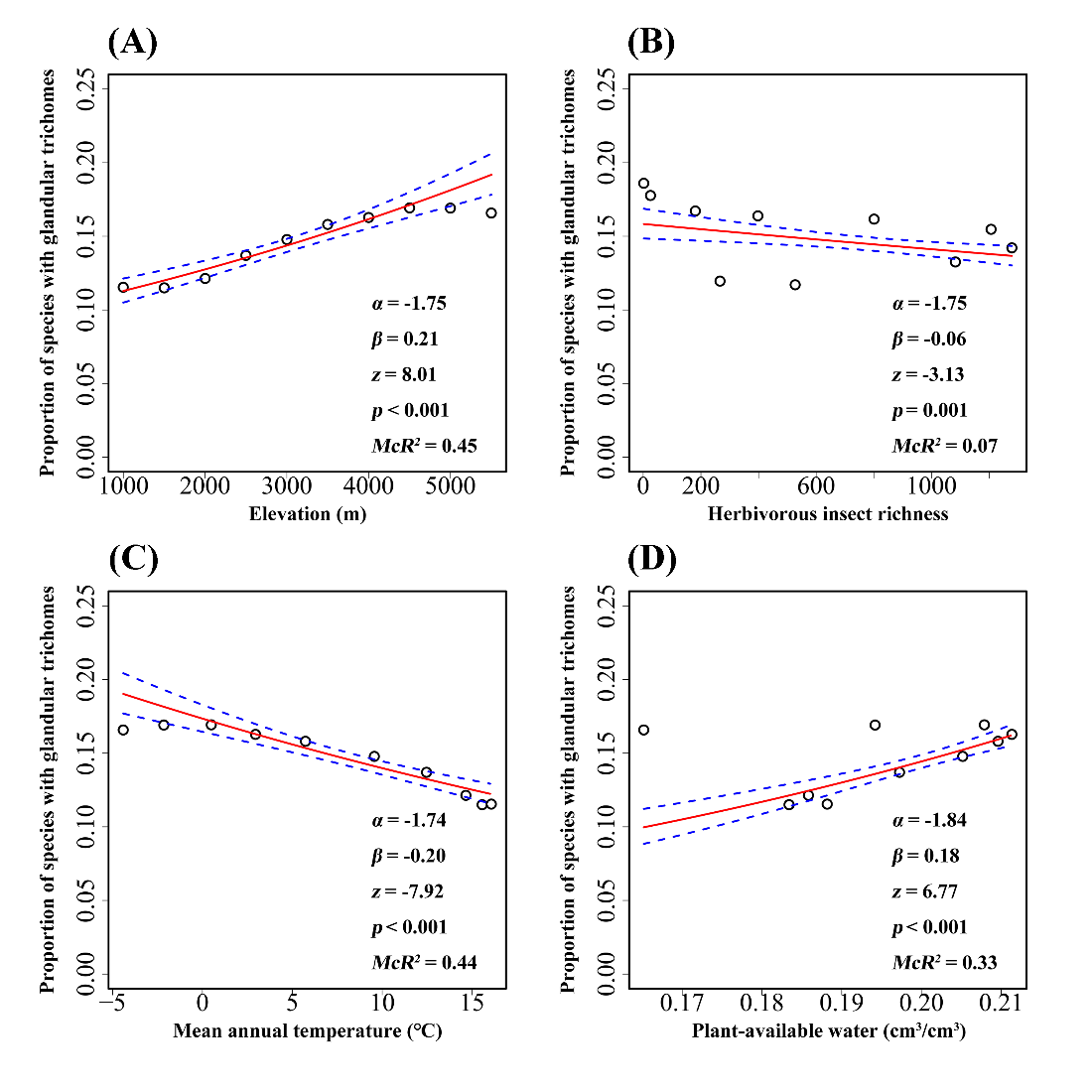


**Fig. S5** Relationship between glandular trichomes and elevation (A), herbivorous insect richness(B), mean annual temperature (C) and plant-available water (D) in the Hengduan Mountains region. The graphs were visualized according to the proportion of plants with glandular trichomes, but the analyses were based on the binary data of presence versus absence of glandular trichomes. The fitted lines (solid line) and estimated 95% confidence intervals (dashed line) display the predicted probability of plants with glandular trichome as fit by logistic regression models. The elevational ranges are from 800 to 5,000 m with every 500 m belt. *α*and *β* values were calculated after predictors being scaled.


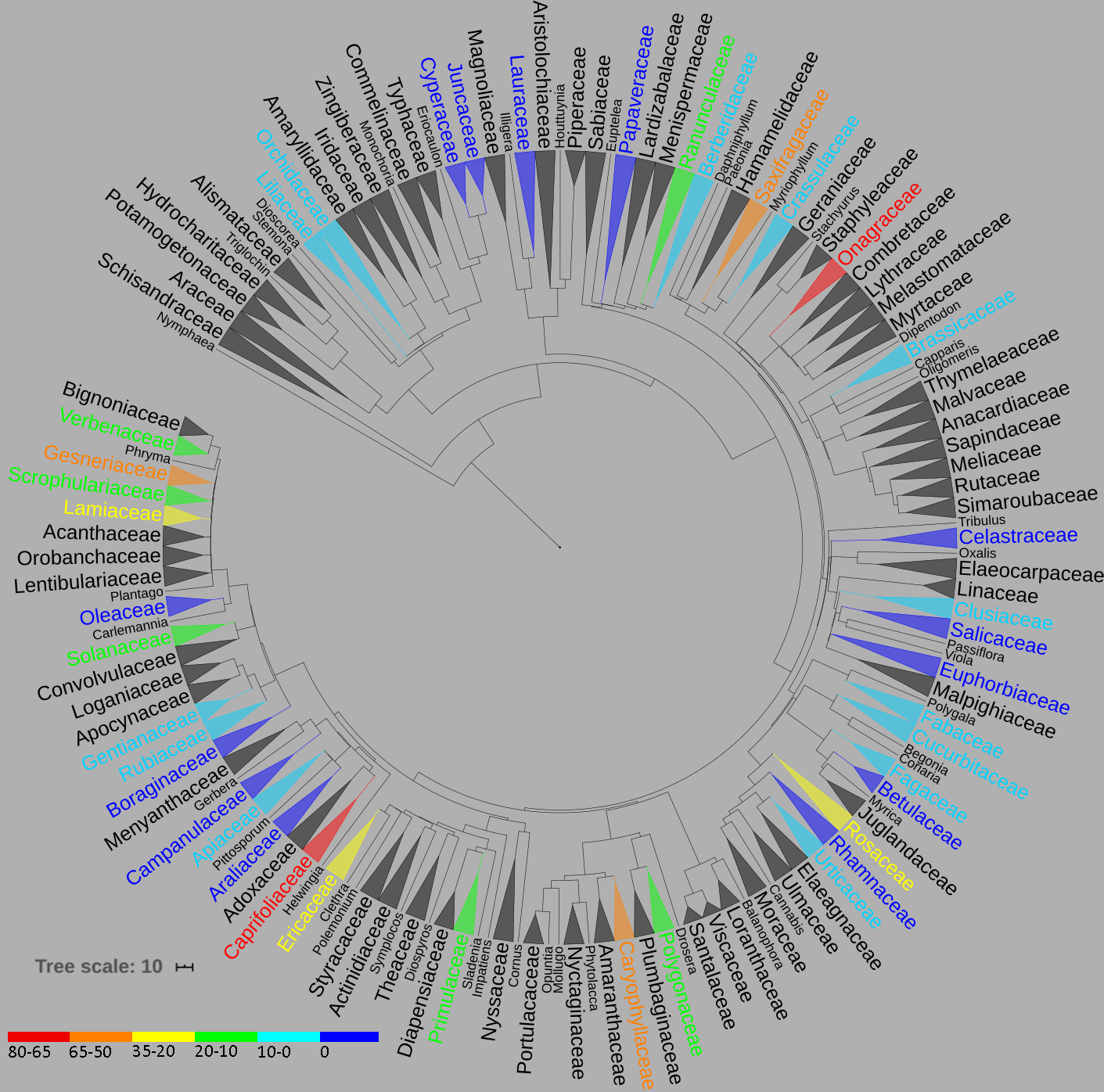


**Fig. S6** Phylogenetic patterns of glandular trichomes. Phylogenetic trees were established by Phylomatic based on Zanne 2014 tree. Colors denote the proportion ranges of plants with glandular trichomes in Families. Families with more than 30 species were colored.


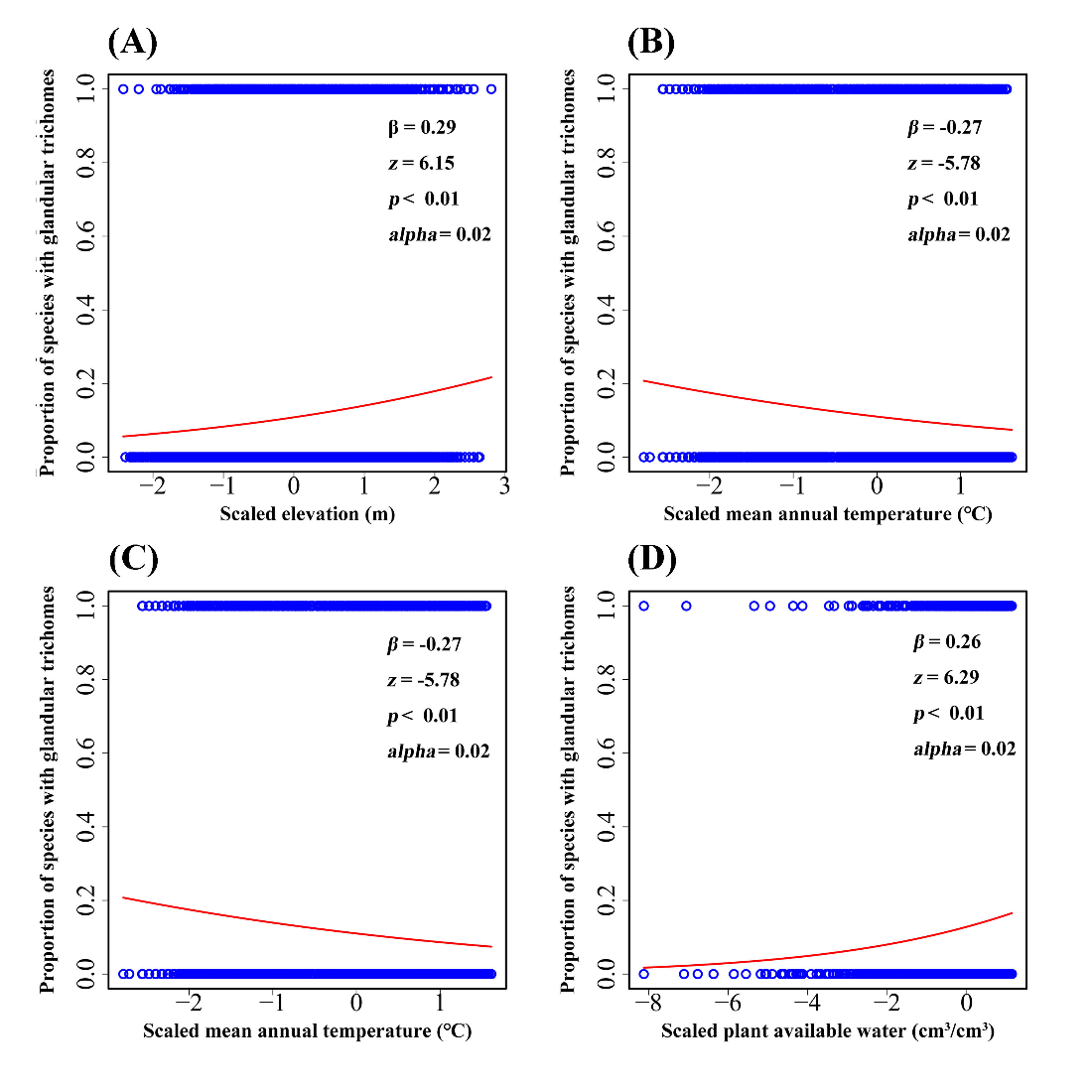


**Fig. S7** Transitions in glandular trichome mode (0 = absence of glandular trichome, 1 = presence of glandular trichome) as functions of (A) elevation, (B) herbivorous insect richness, (C) mean annual temperature and (D) plant-available water. The fitted lines were calculated using phylogenetic logistic regression.
